# Surgery for primary hyperparathyroidism in Germany, Switzerland, and Austria: an analysis of data from the EUROCRINE registry

**DOI:** 10.1007/s00423-023-02819-2

**Published:** 2023-04-13

**Authors:** L. Hargitai, T. Clerici, T. J. Musholt, P. Riss, on behalf of the EUROCRINE council

**Affiliations:** 1 Division of Visceral Surgery, Department of General Surgery, Medical University Vienna, Vienna, Austria; 2 Department of Surgery, Cantonal Hospital St. Gallen, St. Gallen, Switzerland; 3 Department of General and Abdominal Surgery, University Medical Centre of the Johannes Gutenberg University Mainz, Mainz, Germany

**Keywords:** EUROCRINE, Primary hyperparathyroidism, Parathyroid hormone, Intraoperative monitoring, Surgery

## Abstract

**Purpose:**

EUROCRINE is an endocrine surgical register documenting diagnostic processes, indication for surgical treatment, surgical procedures, and outcomes. The purpose was to analyse data for PHPT in German speaking countries regarding differences in clinical presentation, diagnostic workup, and treatment.

**Methods:**

All operations for PHPT performed from 07/2015 to 12/2019 were analysed.

**Results:**

Three thousand two hundred ninety-one patients in Germany (9 centres; 1762 patients), Switzerland (16 centres; 971 patients) and Austria (5 centres; 558 patients) were analysed. Hereditary disease was seen in 36 patients in Germany, 16 patients in Switzerland and 8 patients in Austria. In sporadic disease before primary operation, PET-CT showed the highest sensitivity in all countries. In re-operations, CT and PET-CT achieved the highest sensitivities. The highest sensitivity of IOPTH was seen in Austria (98.1%), followed by Germany (96.4%) and Switzerland (91.3%). Operation methods and mean operative time reached statistical significance (*p*<0.05). Complication rates are low. Overall, 656 (19.9%) patients were asymptomatic; the remainder showed bone manifestations, kidney stones, fatigue and/or neuropsychiatric symptoms.

**Conclusion:**

Early postoperative normocalcaemia ranged between 96.8 and 97.1%. Complication rates are low. PET-CT had the highest sensitivity in all three countries in patients undergoing primary operation as well as in Switzerland and Austria in patients undergoing re-operation. PET-CT could be considered a first-line preoperative imaging modality in patients with inconclusive ultrasound examination. The EUROCRINE registry is a beneficial and comprehensive data source for outcome analysis of endocrine procedures on a supranational level.

## Introduction

Primary hyperparathyroidism (PHPT) is the third most common endocrine disease. Sporadic forms of PHPT, the most common form of PHPT, are most frequently seen in women after the age of 50 [1]. In children and young adults, PHPT is predominantly associated with hereditary diseases, such as multiple endocrine neoplasia (MEN) types 1 and 2a, due to autosomal dominant transferred mutations [1].

With the introduction of intraoperative PTH assays (IOPTH), patients with successful preoperative localisation of the parathyroid adenoma can successfully undergo focused parathyroidectomy (PTX). The advantages of focused parathyroidectomy in relation to a unilateral or bilateral exploration include a shortened operating-time, reduced costs, fewer complications (1–3%), and the patients can be operated on as an outpatient basis [2]. In addition, focused parathyroidectomy has a high success rate of 95-98%, similar to classic PTX [2]. A classic PTX is conducted if localisation diagnostics were negative and if hyperplasia or familial disease is suspected or has been diagnosed [2].

The most employed preoperative localisation imaging studies include neck ultrasound (US) and ^99m^Tc-sestamibi scintigraphy (MIBI). Other localisation studies that can be applied include computed tomography (CT), ^11^C-Methionine PET/CT scintigraphy, ^18^F-Cholin PET-CT, 4D CT, magnetic resonance imaging (MRI) and selective venous sampling with PTH measurement.

To standardise the reporting of diagnostic processes, indication for surgical treatment, surgical procedures and outcomes, EUROCRINE, an international endocrine surgical register based in the EU, was established in 2013. The EUROCRINE database allows for the documentation of operation indication, preoperative diagnostics, perioperative patient management and postoperative follow-up data.

The goal of EUROCRINE is to decrease morbidity and mortality in endocrine tumours, with a particular focus on rare tumours by increasing a surgeon’s awareness of their own outcome-results and enable them to benchmark their results on a national and European level, thus raising clinical standards of care. The data entered into this register is used for data control and can be analysed at the local hospital level, as well as on a national and supranational level. In total, more than 88,000 operations on the thyroid, parathyroid, neuroendocrine tumours, adrenal glands and paragangliomas have been registered in the EUROCRINE database until the end of 2019.

The aim of this study was to analyse EUROCRINE data of patients operated for PHPT in German speaking countries (Germany, Austria and Switzerland) between 2015 and 2019 regarding ethology, clinical presentation, preoperative workup, intraoperative management and outcome. While a study analysing the data of all of the EUROCRINE members regarding sporadic PHPT has been previously published [3], these specific countries were chosen as they use similar preoperative laboratory testing and localisation studies, operation techniques and postoperative routine methods and follow-up.

## Materials and Methods

### Patients

All patients with the biochemical diagnosis of PHPT who underwent parathyroidectomy during the time-period July 2015–December 2019 were included in this analysis. A total of 3,291 patients were included from the following countries: Germany (9 centres; 1762 patients), Switzerland (16 centres; 971 patients) and Austria (5 centres; 558 patients).

Inclusion criteria were complete datasets with information regarding clinical symptomatology, preoperative neck ultrasound and MIBI scan, pre- and postoperative calcium parameters, form of surgery, final histology and available follow-up. All patients signed a written informed consent to all diagnostic and surgical procedures as well as documentation in the EUROCRINE register and data analysis. Documentation regarding the operation and all other required preoperative information (e.g. localisation studies, blood values etc.) are entered into the EUROCRINE register by a member of the surgical team after surgery. All follow-up data is entered into the register when the patient is seen in the outpatient clinic and the data quality and completeness of data is reviewed and verified by the surgeon. The majority of the data entries are standardised by predefined variable lists.

### Statistical analysis

Categorical variables are reported as absolute numbers and percentages, while continuous variables are given as median and first and third quartile. Sensitivity and positive predictive value (PPV) were calculated using 2 × 2 tables. Descriptive statistics analysed patient characteristics. Analysis of data was performed using SPSS version 23 for Windows (Chicago, IL, USA).

## Results

### Patient characteristics

A total of 3291 patients (female: *n*=2506; male: *n*=785) diagnosed with primary hyperparathyroidism were subsequently operated on at 30 different institutions. Sixty patients were diagnosed with hereditary disease: 36 patients (2.0%) in Germany, 16 patients (1.6%) in Switzerland and 8 patients (1.4%) in Austria). Overall, the rate of hereditary disease was 1.82%. In this analysis, age (*p*<0.05), mean preoperative total calcium (*p*<0.05) and mean postoperative total calcium (*p*<0.024) were significantly different between the three countries. Patients’ characteristics are described in detail in Table 1.

**Table 1:** Overall patients’ characteristics and surgical procedures. Categorical variables are reported as absolute numbers and percentages, while continuous variables are given as mean with standard deviation

	Germany	Switzerland	Austria	Total	*p* value
Males:females	414 (12.6):1348 (41.0)	220 (6.7):751 (22.8)	151 (4.6):407 (12.3)	785 (23.9):2506 (76.1)	-
Age (years)	60.2 (± 13.6)	61.9 (± 13.1)	58.1 (± 14.3)	60.4 (± 13.6)	*p*<0.05
Mean preoperative calcium (mmol/l)	2.81 (± 0.23)	2.78 (± 0.24)	2.79 (± 0.25)	2.80 (± 0.22)	*p*<0.05
Mean postoperative calcium (mmol/l)	2.36 (± 0.20)	2.34 (± 0.21)	2.27 (± 0.19)	2.34 (± 0.21)	*p*=0.024
Hereditary disease:				60 (1.82)	-
MEN1	33 (1.0)	15 (0.46)	8 (0.24)	56 (1.7)	
MEN2A	-	1 (0.03)	-	1 (0.03)	
FHPT	3 (0.09)	-	-	3 (0.09)	

### Preoperative imaging in sporadic disease before primary operation

In all three countries, US and sestamibi were the most frequently conducted preoperative imaging modalities, followed by CT and lastly PET-CT. Interestingly, PET-CT achieved the highest sensitivity in all countries: Austria 95.3%, Switzerland 92.5% and Germany 87.5%. While in Switzerland, PET-CT also had the highest PPV of 95.2%, in Austria, it was CT scan with 94.1% and in Germany sestamibi and US (91.8% for both imaging modalities). See Table 2.

**Table 2:** Preoperative imaging in sporadic disease before primary operation. Categorical variables are reported as absolute numbers and percentages as well as 95% confidence intervals

Germany	Sestamibi 1203 (75.8)	US 1468 (92.4)	CT 94 (5.9)	PET-CT 10 (0.6)
TP	814 (67.7)	1091 (74.3)	62 (66.0)	7 (70.0)
FP	73 (6.1)	98 (6.7)	10 (10.6)	2 (20.0)
FN	316 (26.2)	279 (19.0)	22 (23.4)	1 (10.0)
Sensitivity	72.0 (69.3,74.6)	79.6 (77.4,81.7)	73.8 (63.1,82.8)	87.5 (47.4,99.7)
PPV	91.8 (91.5,92.0)	91.8 (91.6,92.0)	86.1 (84.5,87.6)	77.8 (72.9,82.0)
Switzerland	Sestamibi 722 (78.8)	US 892 (97.4)	CT 103 (11.2)	PET-CT 112 (12.2)
TP	496 (68.7)	657 (73.6)	79 (76.7)	99 (88.4)
FP	59 (8.2)	88 (9.9)	5 (4.9)	5 (4.5)
FN	167 (23.1)	147 (16.5)	19 (18.4)	8 (7.1)
Sensitivity	74.8 (71.3,78.1)	81.7 (78.9,84.3)	80.6 (71.4,87.9)	92.5 (85.8,96.7)
PPV	89.4 (88.9,89.8)	88.2 (87.8,88.5)	94.1 (93.5,94.6)	95.2 (94.9,95.4)
Austria	Sestamibi 418 (81.2)	US 481 (93.4)	CT 24 (4.7)	PET-CT 68 (13.2)
TP	299 (71.6)	325 (67.6)	16 (66.6)	61 (89.7)
FP	34 (8.1)	43 (8.9)	1 (4.2)	4 (5.9)
FN	85 (20.3)	113 (23.5)	7 (29.2)	3 (4.4)
Sensitivity	77.9 (73.4,81.9)	74.2 (69.8,78.2)	69.6 (47.1,86.8)	95.3 (86.9,99.0)
PPV	89.8 (89.3,90.3)	88.3 (87.7,88.9)	94.1 (92.4,95.5)	93.9 (93.5,94.2)

*TP*, true positive, *FP*, false positive, *FN*, false negative, *PPV*, positive predictive value

### Preoperative imaging in sporadic disease before re-operation

Similarly, in patients with sporadic disease before re-operation, US and sestamibi were the most frequently conducted preoperative imaging modalities in all three countries. CT scan achieved the highest sensitivity in Germany (84.0%), while in Switzerland and Austria, PET-CT attained the highest sensitivity of 94.1% and 92.9%, respectively. Interestingly, PET-CT had a PPV of 100% in both Germany and Austria, while in Switzerland, CT scan achieved 100% PPV. See Table 3.

**Table 3:** Preoperative imaging in sporadic disease before re-operation*.* Categorical variables are reported as absolute numbers and percentages as well as 95% confidence intervals

Germany	Sestamibi 133 (76.4)	US 144 (82.8)	CT 54 (31.0)	PET-CT 8 (4.6)
TP	88 (66.1)	82 (56.9)	42 (77.8)	4 (50.0)
FP	7 (5.3)	9 (6.3)	4 (7.4)	0 (0)
FN	38 (28.6)	53 (36.8)	8 (14.8)	4 (50.0)
Sensitivity	69.8 (61.0,77.7)	60.7 (52.0,69.0)	84.0 (70.9,92.8)	50.0 (15.7,84.3)
PPV	92.6 (91.8,93.4)	90.1 (88.8,91.3)	91.3 (90.3,92.2)	100 (63.1,100)
Switzerland	Sestamibi 46 (83.6)	US 44 (80.0)	CT 12 (21.8)	PET-CT 19 (34.5)
TP	33 (71.7)	26 (59.1)	8 (66.7)	16 (84.2)
FP	4 (8.7)	4 (9.1)	0 (0)	2 (10.5)
FN	9 (19.6)	14 (31.8)	4 (33.3)	1 (5.3)
Sensitivity	78.6 (63.2,89.7)	65.0 (48.3,79.4)	66.7 (34.9,90.1)	94.1 (71.3,99.9)
PPV	89.2 (87.6,90.6)	86.7 (83.8,89.1)	100 (73.5,100)	88.9 (87.7,90.0)
Austria	Sestamibi 35 (81.4)	US 39 (90.7)	CT 7 (16.3)	PET-CT 14 (32.6)
TP	23 (65.7)	23 (59.0)	1 (14.3)	13 (92.9)
FP	4 (11.4)	2 (5.1)	0 (0)	0 (0)
FN	8 (22.9)	14 (35.9)	6 (85.7)	1 (7.113)
Sensitivity	74.2 (55.4,88.1)	62.2 (44.8,77.5)	14.3 (0.4,57.9)	92.9 (66.1,99.8)
PPV	85.2 (82.4,87.6)	92.0 (89.9,93.7)	100 (59.0,100)	100 (76.8,100)

*TP*, true positive, *FP*, false positive, *FN*, false negative, *PPV*, positive predictive value

### Preoperative imaging in hereditary disease

Also, in patients with hereditary disease, sestamibi and US were the most conducted preoperative imaging modalities. The highest sensitivity of 100% was achieved for CT scan in Germany and Switzerland, while in Austria, it was US with 83.3%. CT and PET-CT scan also had 100% PPV in Switzerland, while in Germany, CT scan achieved 100% PPV and in Austria sestamibi with 83.3%. See Table 4.

**Table 4:** Preoperative imaging in hereditary disease. Categorical variables are reported as absolute numbers and percentages as well as 95% confidence intervals

Germany	Sestamibi 24 (66.7)	US 29 (80.6)	CT 2 (5.6)	PET-CT 0
TP	21 (87.5)	26 (89.7)	2 (100)	-
FP	0 (0)	1 (3.4)	0	-
FN	3 (12.5)	2 (6.9)	0	-
Sensitivity	87.5 (67.6,97.3)	92.9 (76.5,99.1)	100 (15.8,100.0)	-
PPV	100 (85.6,100)	96.3 (95.9,96.7)	100 (15.8,100.0)	-
Switzerland	Sestamibi 14 (87.5)	US 12 (75.0)	CT 2 (12.5)	PET-CT 4 (25.0)
TP	8 (57.1)	8 (66.7)	2 (100)	4 (100)
FP	2 (14.3)	1 (8.3)	0	0
FN	4 (28.6)	3 (25.0)	0	0
Sensitivity	66.7 (34.9,90.1)	72.7 (39.0,94.0)	100 (15.8,100.0)	100 (39.8,100.0)
PPV	80.0 (72.8,85.7)	88.9 (84.8,92.0)	100 (15.8,100.0)	100 (39.8,100.0)
Austria	Sestamibi 8 (100)	US 8 (100)	CT 0	PET-CT 0
TP	5 (62.5)	5 (62.5)	-	-
FP	1 (12.5)	2 (25.0)	-	-
FN	2 (25.0)	1 (12.5)	-	-
Sensitivity	71.4 (29.0,96.3)	83.3 (35.9,99.6)	-	-
PPV	83.3 (75.8,88.9)	71.4 (63.6,78.1)	-	-

*TP*, true positive, *FP*, false positive, *FN*, false negative, *PPV*, positive predictive value

### Symptoms

Overall, 656 (19.9%) patients were asymptomatic. Bone manifestations were seen in 1322 (40.2%) patients, 1128 (34.3%) reported fatigue and/or neuropsychiatric symptoms and 452 (13.7%) kidney stones.

### Surgical procedures and intraoperative PTH monitoring

Mean operation time was the lowest in Germany with 58.5 ±40.6 min, followed by Switzerland (80.8 ±46.4 min) and then Austria (81.2 ±48.4 min). Operation time showed statistical significance between the different countries (*p*<0.05). The highest sensitivity of IOPTH was seen in Austria (98.1%), followed by Germany (96.4%) and then Switzerland (91.3%). Focused parathyroidectomies were performed most often in Switzerland (81.7%), followed by Germany (73%) and then Austria (71.5%). BNE were conducted most frequently in Austria (28.5%) followed by Germany (27.0%) and lastly Switzerland (18.3%). Differences in operation method also achieved statistical significance (*p*<0.05). Overall, 85.6% of patients had a single parathyroid adenoma removed and in 13.4% of patients more than one gland was removed. Multiglandular disease was seen in 13.4% of the patients. The majority of additional thyroid operations were conducted in Austria, where total thyroidectomies occurred in 92 (16.5%) of patients and hemi-thyroidectomies in 105 (18.8%) patients. In Germany, total thyroidectomies were performed in 116 (6.6%) patients and hemi-thyroidectomies in 233 (13.2%) patients. In Switzerland, total thyroidectomies were only carried out in 34 (3.5%) patients and hemi-thyroidectomies in 141 (14.5%) patients. This difference in concomitant thyroid operations also achieved statistical significance (*p*<0.05). Surgical procedures are displayed in Table 5.

**Table 5:** Surgical procedures. Categorical variables are reported as absolute numbers and percentages, while continuous variables are given as mean with standard deviation

	Germany	Switzerland	Austria	Total	*p* value
Mean operative time	58.5 (± 40.9)	80.8 (± 46.4)	81.2 (± 48.4)	68.9±45.3 min	*p*<0.05
IOPTH					-
Yes	1628 (92.4)	845 (87.0)	554 (99.3)	3027 (92.0)	
No	134 (7.6)	126 (13.0)	4 (0.7)	265 (8.0)	
Sensitivity	96.4 (95.3,97.2)	91.3 (89.2,93.0)	98.1 (96.4,99.0)	96.8 (96.1,97.4)	
PPV	99.4 (99.3,99.4)	99.5 (99.5,99.5)	100 (100)	99.5 (99.5,99.5)	
Focused parathyroidectomy	1287 (73.0)	793 (81.7)	399 (71.5)	2479 (75.3)	*p*<0.05
BNE	475 (27.0)	178 (18.3)	159 (28.5)	812 (24.7)	
Number glands removed				3291	-
<1	20 (1.1)	11 (1.1)	3 (0.5)	34 (1.0)	
1	1517 (86.1)	820 (84.5)	478 (85.7)	2815 (85.6)	
>1	225 (12.8)	140 (14.4)	77 (13.8)	442 (13.4)	
Total thyroidectomy: hemi-thyroidectomy	116 (6.6): 233 (13.2)	34 (3.5): 141 (14.5)	92 (16.5): 105 (18.8)	242 (7.4): 479 (14.6)	*p*<0.05

*BNE*, bilateral neck exploration

### Follow-up

## Histology

Of the 3291 patients that underwent surgery, final histology revealed parathyroid cancer in 16 patients (0.5%), six (0.3%) patients in Germany, six (0.6%) patients in Switzerland and four (0.7%) patients in Austria. Parathyroid adenomas and hyperplasia were diagnosed in 3133 (95.2%) patients,. The remaining histology reports revealed normal glands in 25 (0.7%) patients and uncertain histology in nine (0.3%) patients. In 22 (0.7%) patients, the operation was considered a negative exploration, and in 86 (2.6%) patients, no information on the final histology was entered into the EUROCRINE register.

### Postoperative haemorrhage

While post-operative complications are rare, in this patient cohort, haemorrhage or hematoma needing operative revision occurred in 14 (0.8%) patients in Germany, five of which had additional hemi-thyroidectomies. In Switzerland, 11 (1.1%) of patients had post-operative hematoma, one of which had a supplementary hemi-thyroidectomy. Hematoma occurred in seven (1.3%) patients in Austria, two of which had additional hemi-thyroidectomies and one a total thyroidectomy. See Table 6.

**Table 6:** Postoperative morbidity. Categorical variables are reported as absolute numbers and percentages

	Germany	Switzerland	Austria	Total	*p* value
Hematoma	14 (0.8)	11 (1.1)	7 (1.3)	32 (1.0)	-
Transient recurrent laryngeal nerve palsy	43 (2.4)	24 (2.5)	17 (3.0)	84 (2.6)	-
Permanent recurrent laryngeal nerve palsy	12 (0.7)	7 (0.7)	5 (0.9)	24 (0.7)	-
Hypercalcaemia	51 (2.9)	31 (3.2)	17 (3.0)	99 (3.0)	-

### Postoperative oral substitution therapy

Unfortunately, the EUROCRINE database does not provide an input variable specifying calcium and/or vitamin D supplementation therapy at the preoperative stage. While one would expect PHPT patients not to have any preoperative supplementation therapy, postoperative hypocalcaemia based on serum calcium level cannot be properly evaluated in this study. Early postoperative calcium and/or vitamin D therapy for hypocalcaemia was observed in 208 (11.8%) patients in Germany, 70 (7.2%) patients in Switzerland and 63 (11.3%) patients in Austria. At discharge, 429 (24.3%) patients in Germany, 494 (50.9%) patients in Switzerland and 140 (25.1%) patients in Austria were prescribed calcium and/or vitamin D therapy.

### Postoperative hypercalcaemia

The overall postoperative rate of hypercalcaemia was low. In Germany, 51 (2.9%) patients presented with postoperative hypercalcaemia, in Switzerland 31 (3.2%) patients and in Austria 17 (3.0%) patients. See Table 6.

### Recurrent laryngeal nerve palsy

Recurrent laryngeal nerve palsy was observed in 43 (2.4%) patients in Germany, of which 14 (4.1%) had additional hemi-thyroidectomies. Three (7.0%) patients had parathyroid cancer and one (2.3%) papillary thyroid cancer. In Switzerland, 24 (2.5%) patients had postoperative transient nerve palsy, 11 (6.5%) of which had additional hemi-thyroidectomies and one (0.6%) total thyroidectomy. One (4.2%) had additional parathyroid cancer. Transient nerve palsy was seen in 17 (3.0%) patients in Austria, of which six (3.0%) had additional hemi-thyroidectomies and two (1.0%) total thyroidectomies. Two (11.8%) patients had additional papillary thyroid cancer.

At 12 months follow-up, 12 (0.7%) patients demonstrated permanent nerve palsy in Germany, 7 (0.7%) patients in Switzerland and 5 (0.9%) patients in Austria. However, one must take into consideration that in 33 (39.3%) of the 84 patients, no follow-up information regarding nerve palsy was entered into the register. See Table 6.

## Discussion

To our knowledge, this is the first international, multi-centre analysis of PHPT data in all three German speaking countries. All the data was pooled from the EUROCRINE register, thus allowing for a uniform analysis of the patients from various countries and operated on by different surgeons.

### Preoperative imaging

According to the consensus statement of ESES, preoperative imaging should include sestamibi as the first investigation, but US by an experienced investigator can also be conducted [4]. In this analysis, the sensitivity of US in sporadic disease before primary operation ranged between 74.2 and 81.7%, and PPV between 88.2 and 91.8%, both of which lie within the literature rates [1, 3, 5]. In patients with hereditary disease, sensitivity and PPV were high ranging between 72.7–92.9% and 71.4–96.3%, respectively. As expected and due to scar tissue in the neck region, sensitivity and PPV decreased to 60.7–62.2% and 86.7–92%, respectively, in patients who underwent re-operation. US is less expensive, non-invasive and quick, but its sensitivity can also decrease in cases of multiple parathyroid adenomas, parathyroid hyperplasia or concomitant thyroid nodules [6, 7]. It is operator-dependent, and it can be difficult to discriminate between parathyroid adenoma and lymph nodes [8, 9].

The most common preoperative imaging technique is ^99m^Tc-MIBI SPECT/CT. Studies have shown that thyroid adenomas, thyroid nodules and thyroid cancer can result in a higher uptake where washout is not observed, leading to false positive and even false negative results [10]. False negative results can arise from small parathyroid adenomas and ectopic adenomas [11]. A recently published study determined that concomitant thyroid nodules in patients with PHPT, regardless if benign or malignant, did not influence MIBI sensitivity [12]. The sensitivity of MIBI in patients with sporadic disease undergoing primary operation ranged from 72 to 77.9% and PPV 89.4 to 91.8%, which lie within the reported ranges [3, 13, 14]. In patients with hereditary disease, sensitivity was slightly lower ranging between 66.7 and 87.5%; PPV was high between 80 and 100%. As with ultrasound, sensitivity and PPV decreased in patients who underwent re-operation, ranging between 69.8–78.6% and 85.2–92.6%, respectively. Size of the parathyroid adenoma and concomitant thyroid nodules are the most important factors influencing the PPV of sestamibi [13]. This can explain the relatively low sensitivities in this analysis, given that an endemic goitre region causes more thyroid nodules, which can influence sestamibi results. A combination of both US and sestamibi achieved a higher sensitivity, especially for those undergoing primary operation (Germany 75.8%, Switzerland 78.3% and Austria 76.1%) or in patients with hereditary disease (Germany 90.2%, Switzerland 66.7% and Austria 77.4%). A combination of sestamibi and US did not greatly improve sensitivity in patients undergoing re-operation.

Given that EUROCRINE did not discriminate between conventional CT and 4D CT, the reported sensitivity of both modalities lies between 46 and 87% [1, 15]. In this study, a sensitivity of 69.6–80.6% in patients with sporadic disease undergoing primary operation was achieved. This sensitivity decreased to 14.3–66.7% in patients undergoing re-operation. However, PPV was much higher and ranged between 91.3 and 100%. In patients with hereditary disease, sensitivity and PPV were 100%, but this is due to the fact that only four patients (two patients, Germany; two patients, Switzerland) received a pre-operative CT scan. The main advantage of CT is the detection of ectopic adenomas. It must be taken into account that CT is not a primary imaging modality, but only used when US and sestamibi are negative. Some disadvantages include cost and radiation, which is double the radiation dose as with sestamibi [16].

Overall, PET-CT had the highest sensitivity in all three countries in patients undergoing primary operation (Austria 95.3%, Switzerland 92.5% and Germany 87.5%) and in Switzerland (94.1%) and Austria (92.9%) in patients undergoing re-operation, similar to the literature range of 93.7–97% [17, 18]. In Germany, PET-CT only achieved a sensitivity of 50% in patients undergoing re-operation, but one must consider that only eight patients underwent PET-CT. The same applies here to PET-CT that it is not a primary imaging modality. PET-CT has numerous advantages over conventional preoperative imaging including shorter execution times, higher efficiency and lower radiation dose with higher resolution imaging [18–23]. Moreover, PET-CT is the best preoperative imaging modality for identifying ectopic adenomas and less operator dependent than US [19, 24, 25]. While PET-CT is associated with higher costs and not readily available in all hospitals, one must consider that higher initial imaging costs may correlate with higher rates of intraoperative parathyroid localisation, thus subsequently lowering the overall costs of treatment. The results of previous studies and this study reinforce the awareness of PET-CT as a first-line preoperative imaging option [19, 26].

### Patients and symptoms

As seen in this analysis, the majority of patients suffering from PHPT are female and over the age of 60 years, in line with the literature [27]. This analysis showed a statistically significant difference in age between the different countries, which is most likely due to the fact that the mean age in Austria is lower than in Switzerland. According to literature, the cause of PHPT is a single, benign, parathyroid adenoma in over 80% of the patients [28]. This was also observed in the EUROCRINE database. The rate of multiglandular disease observed in this analysis was slightly lower than in the literature 15–20% [28]. The overall rate of hereditary disease is in accordance with a rate of less than 10% cited in the literature [29]. In this patient cohort, parathyroid carcinoma was observed overall in 0.5% of patients, which is a rate similar to what is found in the literature [28].

In the literature, the rate of osteoporosis varies between 39 and 62.9% [30, 31]. Bone manifestations were the most common symptom observed in this study. One must take into consideration that a diagnosis of osteoporosis in older women can also lead to screening for PHPT. Interestingly, 34.3% of patients in this analysis demonstrated fatigue or neuropsychiatric symptoms, which is on the lower end of what is found in the literature (30–62%), but this may be due to the fact that these types of symptoms can be missed in the diagnosis of PHPT, especially in elderly patients [32–34]. Lastly, the rate of patients with kidney stones observed in this study lies within the literature span of 10–20% [35].

### Surgical procedures

In this study, the majority of operations conducted were focused parathyroidectomy followed by BNE. Operation method achieved statistical significance (*p*<0.05). In accordance to the ESES consensus statement, when the abnormal parathyroid gland(s) can be localised pre-operatively, a focused approach is recommended [4]. This results in decreased anaesthesia and operation time, smaller incision size and number of dissections, thus less scar tissue. The early postoperative rate of normocalcaemia was very high: 97.1% in Germany, 96.8% in Switzerland and 97% in Austria. These results lie within the literature and a recently published meta-analysis [36]. However, one must take into consideration that early postoperative normocalcaemia does not equate to cure (defined as normocalcaemia at 6 months after surgery) and that early post-operative recurrences within the first 6 months could still occur and were not part of this analysis.

If one preoperative image (US or ^99m^Tc-sestamibi) is negative, focused exploration with IOPTH measurement or unilateral neck exploration recommended [4, 37]. If both are negative, or discordant, bilateral neck exploration with IOPTH measurement is recommended [4, 37]. In the overall patient population, 623 had a negative sestamibi, of which 326 (52.3%) patients also had negative US. Of these patients, 201 (61.7%) patients underwent a BNE, 71 (21.8%) patients an UNE and 54 (16.5%) patients a focused parathyroidectomy. This is an interesting result, for while the majority of patients did undergo a BNE, 38.3% still underwent UNE or even focused parathyroidectomy; however, the final decision as to which operation is conducted lies with the surgeon. In this analysis, a statistically significant difference was seen between the operation times: Germany had a mean operation time of 58.4 min, which was much lower than Switzerland (80.8 min) and Austria (81.2 min). One explanation for the significant difference in operation times is that some clinics finish the operation and do not wait for the IOPTH end results. Furthermore, total turnover time from blood sampling to final result differs widely between the clinics (according to personal communication).

The majority of additional thyroid operations were conducted in Austria (105 (18.8%) hemi-thyroidectomies and 92 (16.5%) total thyroidectomies) followed by Germany (233 (13.2%) hemi-thyroidectomies and 116 (6.6%) total thyroidectomies) and lastly Switzerland (141 (14.5%) hemi-thyroidectomies and 34 (3.5%) total thyroidectomies). The difference of supplementary thyroid operations achieved statistical significance (*p*<0.05). One can postulate that the patients who underwent hemi-thyroidectomies due to parathyroid disease were either due to intrathyroidal parathyroids or perhaps due to erroneous entries into the EUROCRINE database, given that all of them also had subsequent nodular goitre, papillary carcinoma, or Hashimoto' thyroiditis. Although central Europe is no longer considered an iodine deficiency area due to iodine prophylaxis, it is still an endemic goitre region. It has been shown that thyroid autonomy can occur more often, even when TSH is in the normal range [38]. Moreover, molecular studies have shown that differentiated thyroid cancers (DTC) in Western Europe demonstrate a higher frequency of ras and GSP mutations than DTC cancers in American patients, suggesting that these mutations may play a more significant role in DTC cancers in Western Europe [39].

Overall, IOPTH was conducted in 92.0% patients, which is much higher than a recently published analysis of all EUROCRINE centres [3]. The highest sensitivity and PPV were achieved in Austria, followed by Germany and then Switzerland. The overall success rate of IOPTH for spontaneous PHPT lies in the literature between 97 and 99% [40–42]. This study was able to demonstrate a similar success rate. While 8.0% of operations were performed without IOPTH, this can be due to the fact that IOPTH use is still controversial and may not be available at all institutions. Remarkably, 178 of these operations were in fact focused parathyroidectomy. However, in 108 of these patients, there were positive MIBI/US localisations and in one patient a positive MRT localisation. While studies have shown that focused parathyroidectomy without IOPTH can lead to higher persisting disease[43], the ESES consensus states that IOPTH is of little added value when preoperative localisation is concordant [4]. Thus, some surgeons may not have used IOPTH due to this statement.

### Postoperative morbidity

Overall, this study observed a low hematoma rate of 1.0%, which lies within the reported literature range of 0.1–1.3% [44, 45]. In addition, the rate of transient recurrent laryngeal nerve palsy was 2.6%, which is on the very low end of the literature range 1–30% [46, 47]. Permanent nerve palsy was observed in 0.7% of patients, which also lies within in the literature range of 0.5–5% [46, 47].

Following surgery for primary hyperparathyroidism, transient hypocalcaemia can occur in 15–30% of patients [48, 49]. Early postoperative hypocalcaemia requiring calcium and/or vitamin D therapy during hospital stay was well below this range in all three countries (between 7.2 and 11.8%). At discharge, between 24.3 and 50.9% of the patients received oral calcium and/or vitamin D therapy. One reason for this increase could be due to the fact that recent studies have shown that bone metabolism is profitable [50, 51] in patients with osteopenia/osteoporosis symptoms. Based on the EUROCRINE data structure during the study period, it cannot be differentiated if the prescription was (a) based on a preoperatively prescribed supplementation therapy, (b) a new prescription as postoperative standard routine independent of biochemical or clinical factors, (c) a new prescription only as reserve in case of clinical symptoms after discharge, or (d) a new prescription for postoperative parathyroid insufficiency.

Postoperative hypercalcaemia hinting at an early persistence of the disease was low in all three countries. This could be due to the fact that serum calcium levels in some patients may take longer to reach normal levels. Additionally, early postoperative therapy with calcium can also cause higher serum calcium levels. Lastly, disease persistence can also cause postoperative hypercalcaemia. Long-term follow-up regarding true hypo-/hyperparathyroidism is not available from the registry due to follow-up examinations conducted at different time intervals after surgery.

## Conclusion

Early postoperative normocalcaemia was high and ranged between 96.8 and 97.1%. Complication rates are low. PET-CT had the highest sensitivity in all three countries in patients undergoing primary operation as well as in Switzerland and Austria in patients undergoing re-operation. PET-CT could be considered a first-line preoperative imaging modality in patients with inconclusive ultrasound examination. EUROCRINE is a beneficial and effective platform enabling assessments and comparisons of preoperative workups, operative techniques and early postoperative outcomes on clinic, national and supranational level, as has been demonstrated in this study.

## Data Availability

All data retrieved from the EUROCRINE register.
